# A Retrospective cohort study of Knosp grade reporting of cavernous sinus invasion in pituitary macroadenomas

**DOI:** 10.1007/s11102-026-01683-9

**Published:** 2026-04-29

**Authors:** Kate Spuler, Roland White, Wu Tzen Lim, Jacob Deeb, James McNeil, George Bouras, Alistair Jukes, Stephen Santoreneos, Alkis Psaltis, Sandy Patel, Sunita M. C. De Sousa

**Affiliations:** 1 Adelaide Medical School, (Faculty of Health and Medical Sciences), University of Adelaide, Adelaide, SA Australia; 2 Central Adelaide Local Health Network, (Research Department), Adelaide, SA Australia; 3 Department of Radiology, Royal Adelaide Hospital, Adelaide, SA Australia; 4 Royal Adelaide Hospital, (Endocrine & Metabolic Unit), Adelaide, SA Australia; 5 South Australian Adult Genetics Unit, Royal Adelaide Hospital, Adelaide, SA Australia; 6 Department of Neurosurgery, Royal Adelaide Hospital, Adelaide, SA Australia; 7 Department of Otorhinolaryngology, Royal Adelaide Hospital, Adelaide, SA Australia; 8 Department of Nuclear Medicine, PET and Bone Densitometry, Royal Adelaide Hospital, Adelaide, SA Australia; 9 Department of Surgery, Otolaryngology Head and Neck Surgery, University of Adelaide and the Basil Hetzel Institute for Translational Health Research, Central Adelaide Local Health Network, Adelaide, SA Australia

**Keywords:** Pituitary adenoma, Knosp classification, Cavernous sinus invasion, Pituitary neuroendocrine tumour, Trans-sphenoidal surgery

## Abstract

**Purpose:**

The Knosp grading system classifies the extent of cavernous sinus invasion (CSI) in pituitary adenomas (PAs). Knosp grade is predictive of surgical remission rates and is used in decision-making for the management of these adenomas. This study evaluates the rate and accuracy of Knosp grade reporting of PAs by radiologists.

**Methods:**

This is a retrospective observational study of 100 consecutive patients with pituitary macroadenomas who underwent pituitary MR imaging between March 2023 and March 2025. The rate of CSI reporting in radiologist reports of the scans was determined, and the reported grade was compared with the Knosp grade calculated by the study panel.

**Results:**

Radiologist reports contained a specific comment regarding CSI, or its absence, in 73/100 (73%) of patients. Analysing left and right cavernous sinuses as separate observations, implicit phrasing reporting a specific Knosp grade occurred in 12/200 (6%), and explicit reporting occurred in 10/200 (5%). Analysis of implicit and explicit reporting revealed a statistically significant difference between the reported Knosp grades and study grades (*p* = 0.02). No significant correlation was found between CSI reporting rates and patients undergoing surgery (*p* = 0.179), radiotherapy (*p* = 0.427), or medical therapy (*p* = 0.127) before or at the time of the MRI.

**Conclusion:**

Despite the integral importance of CSI in pituitary adenoma management decisions as enshrined in international guidelines, the rate and accuracy of Knosp grade reporting are suboptimal. Knosp reporting in MRI reports should be standardised to improve the rate and quality of CSI reporting.

## Introduction

Pituitary adenomas (PAs) are typically histologically benign tumours originating from the pituitary gland which may be subclassified as microadenomas (maximal tumour diameter < 10 mm) or macroadenomas (≥ 10 mm). The cavernous sinuses are complex anatomic structures, with vital neurovascular contents, that lie adjacent to the pituitary gland, and are therefore at risk of direct invasion by pituitary macroadenomas. Cavernous sinus invasion (CSI) by pituitary macroadenomas is known to be a major predictor of long-term prognosis [1, 2]. CSI decreases likelihood of successful surgical resection, predicts tumour recurrence and compromises biochemical remission in hypersecretory PAs [1, 3]. Determination of CSI extent is imperative for pre-operative neurosurgical planning of resections as parasellar growth (i.e., whether invading or displacing the cavernous sinuses) may influence whether complete or partial resection will be safely achievable [3]. In a retrospective study of 228 patients diagnosed with PAs, rate of cure following surgery was dependent on degree of CSI (by Knosp classification), with higher grades decreasing rate of cure [6]. Risk of complications also increased with greater CSI [6]. As CSI is generally clinically elusive, accurate radiological assessment is vital.

**Fig. 1:**
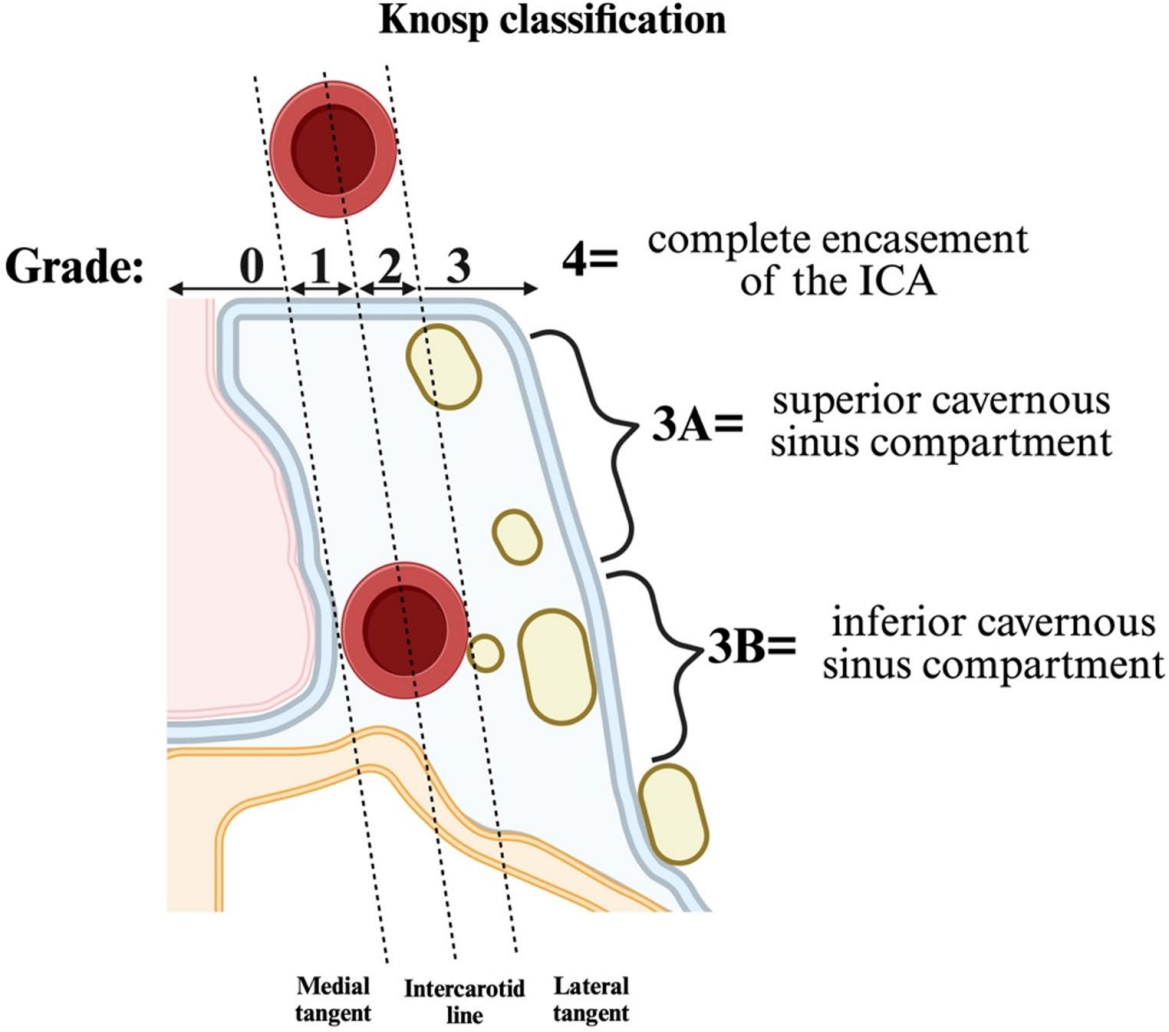
Graphic depicting the boundaries used to determine Knosp Grade in pituitary adenomas. Created using BioRender (Toronto, ON M5V 2J1, Canada)

Knosp grading is a radiological classification of CSI severity that is accurate in predicting the chance of gross-total PA resection and endocrine remission of hypersecretory PAs [3]. The Knosp classification (Fig. 1), which ranges from Grade 0 (no invasion) to Grade 4 (complete circumferential encasement of the ICA), has proven to be an effective tool for predicting surgical outcomes. These grades are allocated based on preoperative coronal MRI of the brain (Fig. 1).

The Knosp grade is determined by lines drawn through the medial margin, centre and lateral margin of the cavernous and supracavernous ICA segments [9]. Recent studies have suggested refining the classification by adding intermediate grades (i.e., Knosp 3 A and 3B) to better differentiate outcomes between tumours invading the superior (Knosp 3 A) versus inferior (Knosp 3B) compartments of the cavernous sinus [7]. Adenomas defined as Grade 3A exhibit a lower rate of invasiveness of the medial cavernous sinus wall than those defined as Grade 3B [7]. This may be related to the different anatomy of the medial wall of the cavernous sinus, which the tumour displaces rather than invading histologically [8].

This study aimed to examine the quality of Knosp grading of CSI in radiology reports in real-world practice, recognising the critical importance of this radiological finding in PA management decision-making.

## Methods

A retrospective observational analysis was conducted on a study cohort consisting of one hundred (*n* = 100) patients diagnosed with pituitary macroadenomas. The inclusion criteria were as follows: (1) patients diagnosed with pituitary macroadenomas, i.e. the tumour was recorded as size ≥ 10 mm at the time of diagnosis, (2) attendance in the Royal Adelaide Hospital (RAH) Pituitary Multi-Disciplinary Team (MDT) Clinic (new or existing patient), (3) aged 18 years or older, (4) MR images and report were available and within study period (March 31, 2023 to March 31, 2025), and (5) tumour present on the Index MRI. Exclusion criteria were: (1) patients without MR imaging showing the cavernous sinuses and relevant anatomical structures for CSI assessment (e.g., no coronal plane), and (2) inadequate quality of MR imaging.

Ethical approval for this study was granted by the Central Adelaide Local Health Network Human Research Ethics Committee (reference number 20728). Due to the nature of the study as a retrospective audit, a waiver of consent to publication was obtained by the Ethics Committee in line with the National Health and Medical Research Council research guidelines.

The South Australian Pituitary Registry was used to identify patients who attended the RAH Pituitary MDT Clinic. Patients diagnosed with macroadenomas were identified; microadenomas and other lesions were excluded. Brain MRI scans for each patient were reviewed to identify the most recent adequate scan that met the inclusion criteria; this scan was termed the Index MRI. Summary statistics were recorded, such as age at diagnosis, age at Index MRI, clinical diagnosis, non-functioning versus functioning macroadenomas, surgery or radiotherapy pre-Index MRI, tumour-targeted medical therapy at time of Index MRI, and histopathological diagnosis (Tables 1 and 3).

The report corresponding to the Index MRI was reviewed. Any comment regarding CSI was documented, as were specific Knosp grades listed. In the case where the report referenced a previous MRI report mentioning CSI, this was classified as the Index report having defined the CSI. Haemorrhage and cystic changes reported by the radiologist were also documented. Histopathology reports were saved; the reported diagnosis was reviewed using the WHO 2022 classification system using hormone and, when available, transcription factor immunohistochemistry.

The Knosp grades reported in the MRI reports were noted. Few reports listed a specific explicit Knosp grade, with many implying Knosp grade through certain phrasing. Phrases implying specific Knosp grades were identified, such as ‘abutment to ICA’, ‘180-degree encasement’, and ‘extension laterally with encasement’ (Table 2). Correlating Knosp grades for each phrase were confirmed by consensus of pituitary specialists. Ambiguous statements where CSI was mentioned but a specific Knosp grade was not indicated were also identified, such as ‘broad abutment of the ICA’, and ‘mild extension into the CS’ (Table 2).

Next, a study panel composed of a neuroradiologist, radiologist and medical graduate determined the Knosp classification grades for the right and left sides of each macroadenoma, independent of any Knosp grade reported in the MRI report. The Knosp grades were determined independently by the medical graduate and the neuroradiologist. The grades were reviewed collaboratively, and the senior neuroradiologist reviewed any cases that were complex or outstanding.

The majority of scans were performed at a single centre, the Royal Adelaide Hospital, which uses a 3 T MRI, with 2.00 mm slice thickness and 0.2 mm gaps. For all studies, T1 sagittal, T1 coronal, and T2 coronal sequences were reviewed and available for optimal visualisation of the cavernous sinuses and coronary arteries. The coronal plane was used in all cases for assessment of CSI invasion.

We next compared the reported grade with our study grade. SPSS Statistics (version 31.0.1.0 (49); IBM Corp., Armonk, NY, USA) and R with ggplot (Wickham H (2016). Springer-Verlag New York. ISBN 978-3-319–24277−4) were used for statistical analysis. A Wilcoxon signed-rank test was used to identify any significant difference between matched data points, with right-sided and left-sided Knosp grades included as separate data points. Chi-square analysis was used to assess for correlation between various factors and CSI reporting rates.

## Results

### Study cohort

The cohort (*n* = 100) consisted of 54% male subjects. Specific summary statistics are featured in Table 1. The age at diagnosis ranged from 17 to 85, with a mean of 50 and a standard deviation of 18. The age at the time of the Index scan ranged from 18 to 89, with a mean of 55 and a standard deviation of 19. All patients were diagnosed with a macroadenoma. 71% of the tumours were non-functioning PAs. The remaining cases (29%) were diagnosed with functioning PAs: 9% acromegaly, 2% Cushing’s disease, 16% prolactinomas, one functioning gonadotrophinoma, and one GH/PRL co-secreting adenoma.

**Table 1:** Summary of patient demographics and summary statistics

Variable	Value (*n* = 100)
Male, number (%)	54 (54)
Age at diagnosis, mean (SD)	50.32 (18.33)
Age at Index MRI, mean (SD)	55.91 (18.70)
Non-functioning adenoma, number (%)	71 (71)
Functioning adenoma, number (%)	29 (29)
Prolactinoma, number (%)	16 (55)
Acromegaly, number (%)	9 (31)
Cushing’s disease, number (%)	2 (7)
Other, number (%)	2 (7)
Surgery prior to Index MRI, number (%)	32 (32)
Radiotherapy prior to Index MRI, number (%)	8 (8)
Tumour-targeted medical therapy at time of Index MRI, number (%)	12 (12)
Index MRI – CSI stated at all^1^, number (%)	73 (73)
Index MRI – Knosp grade implicitly or explicitly reported either side^2^, number, (%)	22 (11)
Index MRI – haemorrhage, number (%)	1 (1)
Index MRI – cystic change, number (%)	14 (14)

^1^Any mention of CSI written in the report

^2^Reports of right and left-sided Knosp Grades taken as individual data points (n=200)

12% of patients underwent surgery prior to the date of the Index MRI. 8% of patients received radiotherapy treatment prior to the Index MRI date. Tumour specific medical therapy (bromocriptine, cabergoline, octreotide or pegvisomant) was received in 12% at the time of the Index MRI.

Baseline tumour diameter at the time of diagnosis ranged from 10 to 58 millimetres (mm). The maximal diameter of tumours at the time of the Index MRI was noted by the reporting radiologist in 64% of cases, with a minimum of 5 mm and a maximum of 44 mm. Radiologist reporting of the tumours revealed haemorrhage in one case and cystic change in 14 cases.

### Knosp grading

Radiologist reports included a comment regarding CSI, or its absence, in 73/100 (73%) of patients. Eight cases (8/100) exhibited explicit Knosp reporting, with 6 cases being non-functioning PA (*n* = 6) and 2 cases being prolactinomas (*n* = 2). When considering left and right cavernous sinuses as separate observations, implicit phrasing reporting a specific Knosp grade was present in 12/200 (6%). Explicit reporting occurred in 10/200 (5%).

Examination of factors such as reporting radiologist, diagnosis, and surgery timing did not reveal any correlation to the frequency of explicit Knosp grade reporting in these eight cases. On two occasions where the Knosp grade was reported explicitly, a range Knosp grading was reported for one side, e.g. ‘Knosp 1 or 2’.

Non-standardised reporting of CSI was prevalent throughout the radiology reports, with various phrases used to describe the degree of CSI (Table 2). Forty-five reports had an implicit Knosp grade range (Table 2). Among these cases, the calculated study grade was within the implicit Knosp grade range in 42 reports (82%).

**Table 2:** Phrases used in radiology reports to describe CSI with correlating Knosp grade or Knosp grade range, were applicable

Phrase	Knosp Grade/Range
Slightly more than 180-degree encasement	Knosp 2
Extension beyond the lateral wall of the cavernous segment of ICA	Knosp 3
Beyond lateral margin of ICA	Knosp 3
Encircles the ICA	Knosp 4
Encasement of the ICA	Knosp 4
Abuts/abutment to ICA	Knosp 0–1
Abutment without extension in the CS	Knosp 0–1
No cavernous sinus invasion	Knosp 0–2
No cavernous sinus involvement	Knosp 0–2
180-degree encasement	Knosp 1–2
Approx. 180-degree encasement of the ICAs	Knosp 1–2
180-degree partial encasement of the ICA	Knosp 1–2
180–270 degree contact with the left supraclinoid ICAs and both A1 segments	Knosp 2–3
Contacts ICAs with < 180 degrees of encasement	Knosp 0–1
Less than 180-degree encasement	Knosp 0–1
Contacts but does not encase cavernous ICA	NA
Broad abutment of the ICA	NA
Lesion within the right CS	NA
Extends into left cavernous sinus	NA
Broad abutment without encasement	NA
Pushing margin into CS	NA
Extending to the left CS	NA
Abutting the ICA with no encasement	NA
Broad contact with the undersurface of the ICA	NA
Pituitary tissue at left CS	NA
Small volume CSI	NA
Lesion within the CS	NA
Contact with the ICA	NA
Stable degree of right ICA abutment	NA
No progressive ICA encasement	NA
Approx 120 degrees of contact	NA
Stable degree of right ICA abutment	NA
Stable appearance of the CS	NA
Abutting approx. 50% of the arteries on both sides	NA
Cavernous sinuses are unremarkable	NA
Contact of pituitary gland with the left CS without invasion	NA
Encases the distal left ICA to its bifurcation	NA

**Fig. 2:**
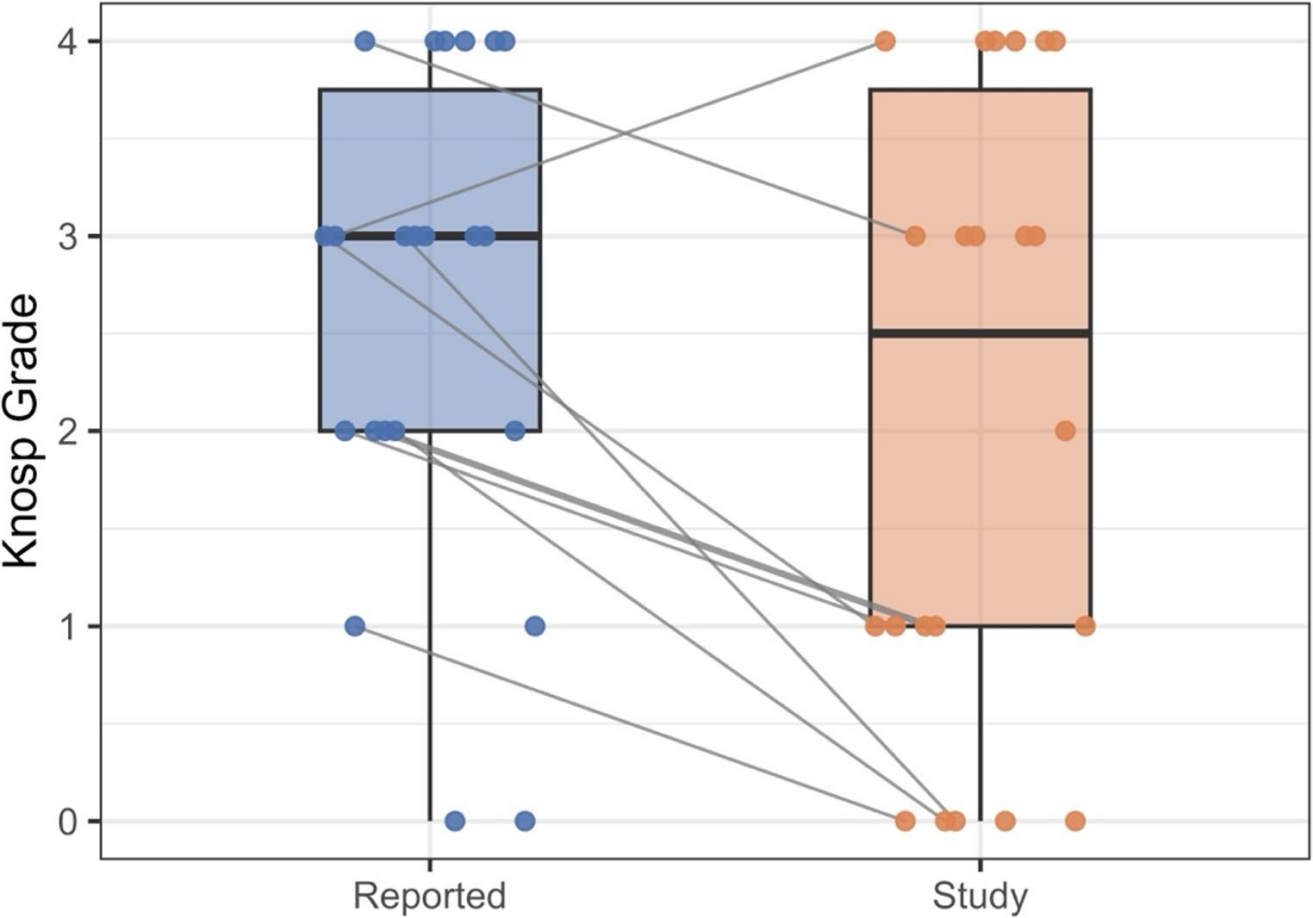
Paired boxplot comparing the reported grade and our study grade. ‘Reported’ grades refer to cases where a specific Knosp grade was implicitly or explicitly reported in the radiology reports. ‘Study’ grades refer to the Knosp Grades calculated in this study for each case

When comparing the reported grading with our calculated study grade, 13 out of the 22 reported grades matched our calculated study grades (59%). Six out of the 10 explicitly reported Knosp grades matched our calculated study grades (60%). A Wilcoxon signed-rank test revealed a statistically significant difference between the reported Knosp grades and our calculated study Knosp grades (*n* = 22; *p* = 0.02), suggesting low accuracy of the reported Knosp grades. The reported Knosp grades were higher (median = 3) compared to the study Knosp grades (median = 2.5), with a moderate effect size (*r* = 0.35). This difference is demonstrated in Fig. 2.

Various factors were analysed against CSI reporting. No significant difference was found between rate of CSI reporting and factors such as whether a patient received surgery prior to the date of Index MRI (*p* = 0.179), radiotherapy prior to (*p* = 0.427), or medical therapy at the time of the Index MRI (*p* = 0.127). The reported diagnosis in the histopathology report was reviewed using the WHO 2022 classification system for this study, using hormone and, when available, transcription factor immunohistochemistry (Table 3).

**Table 3:** Table showing frequency of histological diagnoses in the cohort, including transcription factors and hormone staining

Variable	Value
Histological report diagnosis (*n* = 39)	
Chromophobe	4
Chromophobe null cell	4
Corticotroph adenoma	1
Gonadotroph adenoma	10
Growth hormone adenoma	2
Lactotroph adenoma	1
Necrotic tissue and inflammatory cells	1
Null cell	3
Only cyst material	1
Pituitary adenoma	7
Plurihormonal tumour	1
Somatotroph adenoma	3
Somatotroph adenoma or acidophilic	1
Histopathology Diagnosis WHO 2022	
Corticotroph adenoma	1
Cyst contents	1
Gonadotroph adenoma	15
Immature PIT1 lineage tumour	3
Lactotroph adenoma	1
Null cell	1
Null cell or Gonadotroph adenoma	7
Plurihormonal tumour	2
Somatotroph adenoma	6
Thyrotroph adenoma	1

## Discussion

Knosp classification categorically determines the grade of cavernous sinus invasion of PAs. This study aimed to investigate the rate and accuracy of Knosp classification reporting in brain MRI reports; it is the first published study to our knowledge to examine the rate and accuracy of Knosp reporting by radiologists in real-world practice (Fig. 2).

Radiologist reports included any mention of CSI, or its absence, in the majority (73%) of the cohort. Implicit reporting of Knosp grade occurred in only 12/200 (6%) cavernous sinuses, with explicit reporting of Knosp grade occurring in just 5%. Comparison of the reported Knosp grades with study determined Knosp grades revealed concordant grading in 59% of cases, with a statistically significant difference between reported and study grades. This suggests low accuracy of the reported Knosp grades. Furthermore, ambiguous phrasing in 12% of cases resulted in the inability to interpret a Knosp grade. The discrepancies in Knosp grade might indicate that nonspecific phrasing indicating Knosp grade is not a reliable method of determining Knosp grade. Hence, a standardised approach to Knosp reporting should be considered to improve accuracy.

Heterogeneity in the terminology used to report Knosp grade was prevalent. Paraphrasing with an implicit Knosp grade may be sufficient for pituitary subspecialists, who are the same clinicians likely to view the scan images and deduce the Knosp grade independently. Paraphrasing is likely to be more problematic for generalists seeing patients with PAs with less experience of the Knosp classification system and potentially less inclination to review the MR images and perform Knosp grading independently.

Knosp grade is specifically referred to in the 2023 Pituitary Society prolactinoma guidelines recommendations. Petersenn et al. report that Knosp classification is a better predictor of surgical success than the distinction between microprolactinoma and macroprolactinoma, and is also predictive of surgical remission rates [10]. Critically, Knosp grade dictates the place of surgery in the guidelines treatment algorithm, although this has been the subject of ongoing debate [11, 12]. Per the guidelines, surgical resection of well-circumscribed prolactinomas that are Knosp grade 0 or 1 is considered an equal first-line option compared to medical therapy because it has a ‘high chance of cure, is cost-effective and avoids long-term dopamine agonist treatment’ [10]. By contrast, medical therapy is the preferred first-line treatment option in patients with a low chance of surgical remission predicted by Knosp grade ≥ 2 [10].

Knosp grade also features in the 2025 Pituitary Society incidentaloma guidelines recommendations. Fleseriu et al. recommend that standardised MRI reports should include a modified Knosp classification, and that incidental macroadenomas with invasive features such as CSI may require closer imaging intervals. Macroadenomas that are enlarging or invading the cavernous sinus may be considered for surgical intervention [13].

Meta-analysis of several studies has confirmed that Knosp grading is the best objective measure for CSI diagnosis in PAs [4, 5]. Compared to other CSI classifications such as percentage encasement of the internal carotid (PEICA), venous compartment obliteration (VCO) and Fernandez-Miranda classification, Knosp grade of ≥ 1 had the highest sensitivity in a 2021 study assessing 210 cavernous sinuses with comparison to the reference assessment of intraoperative findings and postoperative day 1 MRI [4]. Other parameters with higher specificity, such as PEICA, may be used in tandem with Knosp classification [4]. Several technical factors are important for the diagnosis of CSI. Firstly, MRI is the gold standard for evaluating CSI in PAs, with studies demonstrating 7-Tesla MRI leading to more reliable assessment of CSI, although this technology is not universally available [8]. Secondly, regarding the surgical diagnosis of CSI, endoscopic techniques provide a more accurate assessment of the cavernous sinus over microscopy [5]. Both radiological and surgical techniques for evaluating CSI have been found to be valuable in predicting prognosis of PAs [2].

A potential limitation of this study is the timing of the Index MRI compared to the time of diagnosis. In some cases, the radiologist may have exercised discretion and not referenced minor CS extension due to a lack of clinical relevance. Another limitation of the retrospective case series is that the mean time between diagnosis and index MRI was > 5.5 years, meaning that in many cases the Index MRI was not the scan taken at diagnosis. However, some scans referenced were dated after surgical resection and therefore a recalculation of the Knosp grade is relevant. Larger, multicentre studies are required to better understand the rate and quality of Knosp grading in radiology reports internationally and investigate potential variability in reporting tendencies among other specialists, such as neurosurgeons, ENT surgeons and endocrinologists. These data may then form the basis of prospective trials of standardised CSI reporting and effects on PA management – particularly outside quaternary pituitary centres, noting that multiple international PA management guidelines necessitate knowledge of the presence and degree of CSI to enact correct assessment and management pathways. The implementation of updated guidelines including Knosp Grade reporting for PAs, with additional training for general radiologists, may assist in standardising Knosp reporting by radiologists and improve accuracy.

In conclusion, noting the suboptimal Knosp grade reporting observed in this cohort and given the prominence of Knosp grade in PA management guidelines, we recommend a standardised approach to reporting of Knosp grade.

## Data Availability

No datasets were generated or analysed during the current study.

## References

[1] Lefevre E, Chasseloup F, Hage M, Chanson P, Buchfelder M, Kamenický P (2024), May 18 Clinical and therapeutic implications of cavernous sinus invasion in pituitary adenomas. Endocrine 85:1058–1065 10.1007/s12020-024-03877-2 38761347

[2] Lu L, Wan X, Xu Y, Chen J, Shu K, Lei T (2022) Classifying Pituitary Adenoma Invasiveness Based on Radiological, Surgical and Histological Features: A Retrospective Assessment of 903 Cases. 11(9) 10.3390/jcm11092464 PMC9104472 35566590

[3] Micko AS, Wöhrer A, Wolfsberger S, Knosp E (2015) Invasion of the cavernous sinus space in pituitary adenomas: endoscopic verification and its correlation with an MRI-based classification. J Neurosurg 122(4):803–811 25658782 10.3171/2014.12.JNS141083

[4] Chang N, Grayson WJ, Mangussi-Gomes J, Jonker PB, McCormack A, Harvey RJ (2021) Assessment of magnetic resonance imaging criteria for the diagnosis of cavernous sinus invasion by pituitary tumors. J Clin Neurosci. 10.1016/j.jocn.2021.06.010 34275561 10.1016/j.jocn.2021.06.010

[5] Dhandapani S, Singh H, Negm MH, Cohen S, Anand KV, Schwartz HT (2016) Cavernous Sinus invasion in Pituitary Adenomas: systematic review and pooled data meta-analysis of radiologic criteria and comparison of endoscopic and microscopic surgery. World Neurosurg 96:36–46 27591098 10.1016/j.wneu.2016.08.088

[6] Araujo-Castro M, Cancela AA, Vior C, Pascual-Corrales E, Berrocal VR (2022), January Radiological Knosp, Revised-Knosp, and Hardy–Wilson Classifications for the Prediction of Surgical Outcomes in the Endoscopic Endonasal Surgery of Pituitary Adenomas: Study of 228 Cases. Front Oncol 20(11) 10.3389/fonc.2021.807040 PMC8810816 35127519

[7] Micko A, Oberndorfer J, Weninger WJ, Vila G, Höftberger R, Wolfsberger S, Knosp E (2019), May 31 Challenging Knosp high-grade pituitary adenomas. J Neurosurg 132(6):1739–1746 31151112 10.3171/2019.3.JNS19367

[8] Serioli S, Doglietto F, Fiorindi A, Biroli A, Mattavelli D, Buffoli B (2019) Poliani. Pituitary Adenomas and Invasiveness from Anatomo-Surgical, Radiological, and Histological Perspectives: A Systematic Literature Review. Cancers 11(12):1936 10.3390/cancers11121936 PMC6966643 31817110

[9] Eisenhut F, Schmidt MA, Buchfelder M, Doerfler A, Schlaffer S-M (2022), December Improved Detection of Cavernous Sinus Invasion of Pituitary Macroadenomas with Ultra-High-Field 7 T MRI. Life 13(1) 10.3390/life13010049 PMC9867165 36675998

[10] Petersenn S, Fleseriu M, Casanueva FF, Giustina A, Biermasz N, Biller MB, Bronstein M (2023), September 5 Diagnosis and management of prolactin-secreting pituitary adenomas: a Pituitary Society international Consensus Statement. Nat Reviews Endocrinol 19(12):722–740 10.1038/s41574-023-00886-5 37670148

[11] Wu ZB (2024), January 22 The shift of therapeutic strategy for prolactinomas: surgery as the first-line option. Nat Reviews Endocrinol 20*:*310 10.1038/s41574-024-00953-5 38253861

[12] Santoreneos S, Wormald P-J, Shivalingam B, Chapman IM, Candy NG, Jukes AK, De Sousa SM (2024), March 20 Tumour fibrosis in dopamine agonist-exposed prolactinomas is a diminishing concern. Nat Reviews Endocrinol 20:314 10.1038/s41574-024-00976-y 38509199

[13] Fleseriu M, Gurnell M, McCormack A, Fukuoka H, Glezer A (2025), June 24 Pituitary incidentaloma: a Pituitary Society international consensus guideline statement. Nat Reviews Endocrinol 21:638–655 10.1038/s41574-025-01134-8 40555795

